# The Similarity Between Chinese Five-Pattern and Eysenck’s Personality Traits: Evidence From Theory and Resting-State fMRI

**DOI:** 10.3389/fnhum.2020.00038

**Published:** 2020-02-13

**Authors:** WenTao Zhao, LiPing Song, Jian Du, XiaoZhen Li, Hao Wang, Long Cheng, Jing Li, Liang Zhang, XinRong Li, QiuLi Yang, Yong Xu

**Affiliations:** ^1^Department of Psychiatry, First Hospital/First Clinical Medical College of Shanxi Medical University, Taiyuan, China; ^2^Department of Humanities and Social Science, Shanxi Medical University, Taiyuan, China; ^3^Institute of Basic Research in Clinical Medicine, China Academy of Chinese Medical Sciences, Beijing, China

**Keywords:** chinese five-pattern personality traits, Eysenck’s personality traits, regional homogeneity, regression analysis, mediation analysis

## Abstract

Chinese five-pattern and Eysenck’s personality traits are two types of personality theories based on different cultural backgrounds. The former is an indigenous theory, and the latter is a cross-cultural theory. In order to verify the relationship between two different personality traits from theory and neuropsychology, the current study recruited 170 healthy adults to calculate their five-Pattern Personality Inventory (FPPI) and Eysenck Personality Questionnaire-Revised (EPQ) scales and to scan their brains using functional magnetic resonance imaging (fMRI). Then, we performed stepwise-regression analysis and mediation-effect analysis to explore the association between brain regional homogeneity (ReHo) and two types of personality traits. The results showed that the ReHo of the right superior temporal gyrus (STG) positively correlated with TaiYang traits for FPPI and that there was a significant linear relationship with extraversion and neuroticism for EPQ. Besides, the ReHo of the right medial prefrontal cortex (mPFC) positively correlated with TaiYin for FPPI, and it also showed a significant linear relationship with neuroticism for EPQ. Furthermore, we found that extroversion and neuroticism partially mediated the relationship between five-pattern personality traits and the regional brain function, based on the mediation-effect analysis. Our findings suggest that Chinese five-pattern personality traits have a close relationship with Eysenck’s personality traits and that both may be engaged in similar neurobiological mechanisms in common brain regions to some extent. Hence, these findings first reveal a relationship between Chinese traditional personality traits and Western Eysenck’s personality traits in terms of both theoretical and neurobiological contexts.

## Introduction

Personality traits are core concepts of psychology that organically integrate into an individual’s specific patterns of feelings, behaviors, emotions, cognition, and thought. Moreover, personality traits determine an individual’s lifestyle and adaptation to the environment (Campbell, 1926; Hershberger, 1994; Pang et al., 2015). Over the past half-century, the instrumental role of neurophysiology—represented by Eysenck’s theory in describing neurophysiological causes of individual personalities—has been fruitfully investigated in the domain of Western psychology. Several personality studies focused on neurobiology have indicated that the neural mechanisms of neuroticism, extroversion, and psychoticism are common psychological and behavioral characteristics that are prevalent in individuals across various cultural backgrounds (Eysenck and Abdel-Khalek, 1989; Loo, 1995; Eysenck and Barrett, 2013).

However, despite the common consensus that Eysenck’s personality traits are cross-cultural, a subset of psychologists with a multicultural view still doubt whether Eysenck’s personality traits can objectively reflect personality characteristics without culture-specific impacts (Avdeyeva and Church, 2005; Schmitt et al., 2007). Therefore, many psychologists have recently been working on constructing indigenous personality theories relevant to culture-specific experiences, such as in India, the Philippines, Korea, and Japan (Guanzon-Lapeña et al., 1998; Chae and Lee, 2015; De Fruyt et al., 2015).

In China, the theory of five-pattern personality traits, which originated from traditional Chinese medicine (TCM) and the ancient philosophy of Yin and Yang, has been viewed as the most culturally characteristic theory for classifying individuals into the five patterns of TaiYang, ShaoYang, PingHe, ShaoYin, and TaiYin based on Yin-Yang differences. The Five-Pattern Personality Inventory (FPPI), a standardized personality scale based on this theory, was established at the China Academy of Traditional Chinese Medicine in 1986 (Li et al., 2015). The concept of Yin and Yang determines individual characteristics of personality according to the theory of TCM. Yang denotes excitement and extroversion, while Yin denotes inhibition and introversion. Besides, the concept of Tai and Shao, representing emotional instability-stability, denotes the abstraction in the quantities of Yang and Yin. Such a description is similar to Eysenck’s personality theory, which based on the excitation-inhibition process in terms of neural activity. Compared with Eysenck’s theory, Ren (2014) considered that five-pattern personality theory should not only contain extraversion, neuroticism, and psychoticism but offered that individuals be further subdivided based on additional characteristics. It is noteworthy that PingHe is a particular conception in the five-pattern personality theory that represents a comprehensive personality that is characterized by balance.

Although Chinese five-pattern and Eysenck’s personality traits are similar in terms of their theoretical descriptions, there is a dearth of literature indicating whether or not they are empirically associated. In recent years, resting-state functional magnetic resonance imaging (rs-fMRI) has been used to further explore the neurobiological basis of personality traits especially extraversion and neuroticism (DeYoung et al., 2010; Adelstein et al., 2011; Kunisato et al., 2011; Wei et al., 2012). Regional homogeneity (ReHo), which represents the level of synchronous activation across brain regions *via* calculation of Kendall’s coefficient of concordance (KCC) of voxels (Zang et al., 2004), was recently adopted to demonstrate a statistical association between Eysenck’s personality traits and brain function (Song et al., 2015). To follow-up on this finding, the purpose of the current study was to investigate whether Eysenck’s personality traits and five-pattern personality traits are engaged in overlapping theoretical structures and neurobiological mechanisms. To quantify and statistically test the degree of these associations, we applied regression analysis combined with ReHo to assess the relationships among FPPI and Eysenck Personality Questionnaire-Revised (EPQ) scores and brain function. Furthermore, we performed the mediation-effect analysis to explore whether Chinese five-pattern traits take effect on the regional brain function by Eysenck’s personality traits. We attempted to explain the relationship, both theoretically and neurobiologically, between two personality traits based on different cultural backgrounds.

## Materials and Methods

### Participants

We recruited 184 right-handed healthy adults from Shanxi Medical University and the surrounding community. The inclusion criteria were as follows: (1) age from 18 to 55 years old; (2) no formal psychotic or substance abuse history; (3) no physical diseases; and (4) no metal implants. Exclusion criteria for the participants included the following: (1) met the criteria for any mental disorder according to the DSM-IV; (2) family psychiatric or substance abuse history; or (3) unsuitable for fMRI scans. Finally, there nine participants excluded as lying subscale in FPPI were >4 and five excluded as excessive head movements parameters, and 170 participants (50 males and 120 females; range = 18–55 years old; mean ± SD = 27.06 ± 8.1 years old) were included in the present study. All of the participants were screened with Symptom Checklist-90 (SCL-90) to ensure that they had no psychiatric or neurological illnesses. After signing the consent form, each participant was required to have an fMRI scan and answered a series of personality-related questions. Participants received payment for their time. This study was approved by the Ethics Committee of Shanxi Medical University.

### Personality Assessments

We separately administered the self-report versions of the FPPI and EPQ. The FPPI consists of 103 items that include five subscales of TaiYang, ShaoYang, TaiYin, ShaoYin, and PingHe. Subjects responded to each item in the questionnaire as “yes” (score = 1) or “no” (score = 0). Besides, the subscale of lying is another personality dimension used to measure the truthfulness of subject responses. If scores of this subscale were >4, the questionnaire was regarded as invalid and excluded from the study. In the present sample, the mean scores of TaiYang, ShaoYang, TaiYin, ShaoYin, and PingHe were 10.57 (SD = 3.74), 12.24 (SD = 3.81), 9.34 (SD = 4.68), 13.90 (SD = 3.32), and 6.56 (SD = 2.55), respectively. These scales have been validated in the Chinese population with high reliability and validity (Wang et al., 2016). The EPQ is designed to assess personality dimensions of extraversion (E), neuroticism (N), and psychoticism (P). In the present sample, the mean scores of E, N, and P were 55.06 (SD = 10.37), 49.81 (SD = 11.98), and 48.92 (SD = 10.40), respectively. These scales have been validated in the Chinese population with high reliability and validity (Qian et al., 2000). Table 1 shows the details of the two personality inventories.

**Table 1:** Demographic and personality characteristics of participants (*n* = 170).

Characteristics	Data	Range
Age (years)	27.06 ± 8.12	18–56
Sex (female/male)	120/50	
TaiYang scores	10.57 ± 3.74	2–20
TaiYin scores	9.34 ± 4.68	0–20
ShaoYang scores	12.24 ± 3.81	2–20
ShaoYin scores	13.90 ± 3.32	2–20
PingHe scores	6.56 ± 2.55	0–10
Extraversion scores	55.06 ± 10.37	35–75
Neuroticism scores	49.81 ± 11.98	25–75
Psychoticism scores	48.92 ± 10.40	30–80

*The data are shown as the mean ± SD*.

### fMRI Acquisition

All of the fMRI scans were collected with a Siemens 3.0-T scanner (Siemens Healthcare GmbH, Erlangen, Germany) at Shanxi Provincial People’s Hospital. A standard eight-channel phase-array head coil was employed and image artifacts, such a head motion and scanner noise, were reduced with foam padding and headphones. During fMRI scanning, all of the participants were required to remain quiet with their eyes closed. The rs-fMRI scans used echo-planar imaging (EPI) pulse sequence and had the following parameters: 32 slices; TR of 2,500 ms; TE of 40 ms; FA of 90°; matrix of 64 × 64; voxel size of 3 × 3 × 3 mm^3^; FOV of 240 × 240 mm; and 212 volume.

### Image Pre-processing

Image processing was carried out using the toolbox for Data Processing and Analysis of Brain Imaging (DPABI) V 3.0 (Yan et al., 2016) in MATLAB 2012a. Briefly, the first 10 volumes of functional images were discarded to ensure signal stability and patient acclimation. The remaining 202 functional scans were slice-acquisition corrected and realigned to account for head motion. Data from participants with head movements exceeding 2.0 mm of maximum displacement in x, y, or z directions or with angular head motion exceeding 2.0° were excluded from further analysis. Subsequently, we regressed out nuisance signals, including signals of the whole brain, white matter (WM), cerebrospinal fluid (CSF), Friston’s 24-parameter motion model (Friston et al., 1996) of head motion. Considering the possible effect of micromovements on fMRI data (Power et al., 2012), the head motions were measured using framewise displacement (FD) values with Power formula for each subject. The mean FD < 0.5 mm were ensured in all subject. Then, the fMRI scans were normalized to the standard SPM8 EPI template and resampled to 3 × 3 × 3 mm^3^. The resulting fMRI data were temporally band-pass filtered (0.01–0.08 Hz) and linearly detrended to reduce low-frequency drifting and high-frequency physiological noise.

ReHo was performed using REST software (Song et al., 2011). The formula used to calculate the KCC value has been reported previously (Zang et al., 2004). Normalization of ReHo maps was accomplished by dividing the KCC for each voxel by the averaged KCC across the entire brain.

### Correlation Analysis

To investigate the correlation between FPPI scores and ReHo values, the voxel-based whole-brain ReHo maps were compared with scale scores by correlation analysis using the DPABI toolbox. Age, gender and mean relative displacement were treated as covariates to control for their individual effects. The significance level was set at *p* < 0.05 and corrected for multiple comparisons with a cluster *p* < 0.05 and voxel *p* < 0. 001, according to Gaussian random field theory (GRF).

### Stepwise Linear Regression Analysis

Based on the above results, we constructed a regression model to investigate further whether the regional brain ReHo values, which were associated with FPPI scores, would show a significant linear relationship between neuroticism, extroversion, and psychoticism scores for EPQ. In this model, the independent variable consisted of the ReHo values of regional brain areas, which were found to be significantly related to FPPI scores in the previous step. The dependent variable consisted of the standardized scores of each subscale of EPQ.

Stepwise linear regression was performed by using SPSS 19.0. Neuroticism, extroversion, and psychoticism scores of EPQ entered into the regression model deferred the forward method step by step. The stepwise selection process was set to a *p*-value of 0.05.

### Mediation Analysis

To examine how extraversion and neuroticism impact the relationships among the five-pattern personality traits and individual differences in regional brain function, a mediation analysis was performed by the PROCESS procedure for SPSS macros. We set up two mediation models from the regression results mentioned above. In the first model, we examined the mediation effect of extraversion and neuroticism in terms of the relationship between the ReHo of the right superior temporal gyrus (STG) and TaiYang scores. In the second model, we examined the mediation effect of neuroticism in terms of the relationship between the ReHo of the right medial prefrontal cortex (mPFC) and TaiYin scores.

Estimates of all of the paths were computed using ordinary least squares (OLS) regression. An indirect effect and direct effect were considered to be significant if the 95% bootstrap confidence intervals (CIs) from 5,000 bootstrap samples did not include zero.

## Results

### Subjects and Personality Scores

The demographics and personality scores of the included subjects are shown in Table 1. The correlations between EPQ and FPPI scores are shown in Table 2.

**Table 2:** Correlation between five-factor personality traits and Eysenck’s personality traits.

Characteristics	Extraversion	Neuroticism	Psychoticism
TaiYang	0.371**	0.011	0.058
TaiYin	−0.422**	0.688**	0.390**
ShaoYang	0.503**	−0.042	0.006
ShaoYin	−0.197*	−0.091	−0.256**
PingHe	0.196*	−0.329**	−0.348**

*Indicates *p* < 0.05. **Indicates *p* < 0.01.

### Correlation Analysis: FPPI and ReHo Values

**Figure 1:**
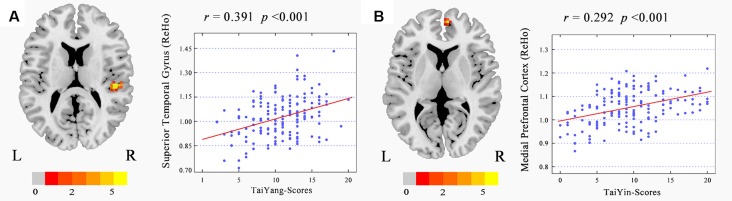
Correlation maps between regional homogeneity (ReHo) value and five-pattern personality inventory (FPPI) scores (GRF corrected, cluster* p* < 0.05 and voxel *p* < 0.001). The red-light color indicates a positive correlation. Brighter dot delegate higher correlations. **(A)** TaiYang scores was positively associated with the ReHo of the right superior temporal gyrus (STG). **(B)** TaiYin scores was positively associated with the ReHo of the right medial prefrontal cortex (mPFC).

The correlation analysis results showed that the ReHo of the right STG was significantly positively correlated to TaiYang scores after controlling for each subject’s age, gender, and mean relative displacement (*r* = 0.391, *p* < 0.0001, cluster size = 30 voxels). The ReHo of the right mPFC was significantly positively correlated to TaiYin scores after controlling for each subject’s age, gender, and mean relative displacement (*r* = 0.292, *p* < 0.0001, cluster size = 22 voxels, see Table 3, Figure 1). No other significant correlations were found for any other dimensions of personality traits of FPPI.

**Table 3:** Significant correlations between personality traits and regional homogeneities.

Personality traits	Brain region	BA area	R/L	MNI coordinates (x, y, z)	Voxels	*t*-value
TaiYang	STG	BA 48	Right	45, −27, 15	30	5.515**
TaiYin	mPFC	BA 10	Right	6, 57, 6	22	3.960**

**Indicates p < 0.01.

### Stepwise Linear Regression Analysis: ReHo Values and EPQ

The results of the stepwise linear regression analysis are summarized in the Supplementary Table S1. Neuroticism and extroversion were retained in the regression model of the right STG (*R*^2^ = 0.170, *p* < 0.001). Neuroticism was retained in the regression model of the right mPFC (*R*^2^ = 0.111, *p* < 0.001).

### Mediation Analysis: Two Types of Personality Traits and ReHo Values

**Figure 2:**
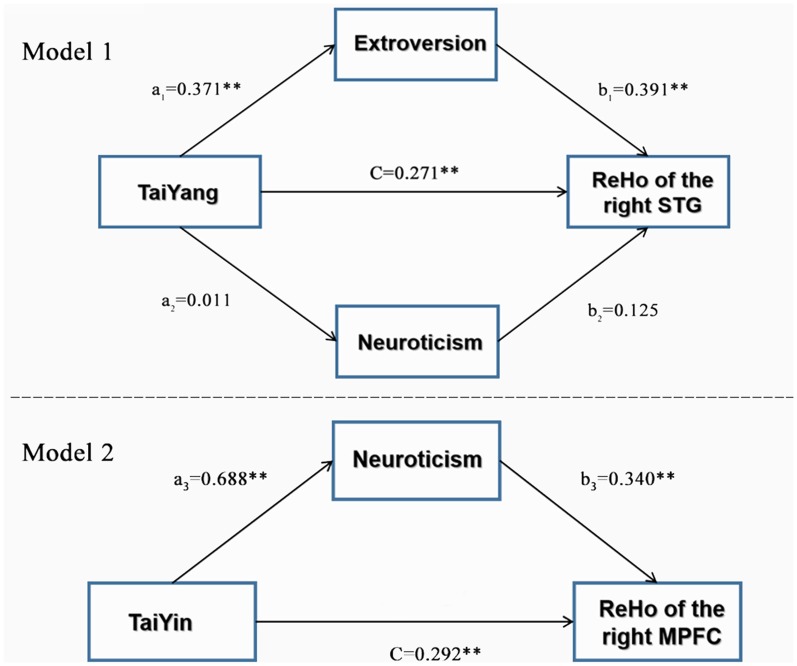
Path linking the five-pattern personality traits to brain function *via* Eysenck’s personality traits. **p < 0.01.

We examined the total effect, direct effect, and indirect effect separately in the two models (Figure 2). In the first mediation model, the total effect between TaiYang and ReHo of the right STG was significant [CI = (0.008, 0.017)]. Further analysis revealed that the direct effect [CI = (0.0043, 0.0135)] and indirect effect [CI = (0.0015, 0.0065)] of extroversion were both significant, as indicated by their CIs that did not contain zero. However, the indirect effect of neuroticism was not significant [CI = (−0.0007, 0.0009)]. This finding indicated that only extroversion played a mediating effect between the TaiYang trait and the ReHo of the right STG.

In the second model, we used the same procedure to examine the total, direct, and indirect effects on the association between the TaiYin scores and the ReHo of the right mPFC as mediated by neuroticism. The result showed a significant indirect effect of neuroticism scores [CI = (0.0009, 0.0066)], but the direct effect were not significant [CI = (−0.0018, 0.0063)]. This finding indicated that neuroticism might also be a mediator between TaiYin traits and the ReHo of the right mPFC. Table 4 and Supplementary Table S2 contains the details of the parameters of these results.

**Table 4:** The indirect effect, direct effect, and total effect between regional homogeneity (ReHo) values and personality traits.

	Effect	BootSE	Bootstrapping BC 95% CI		Relative effects
			Lower limit	Upper limit	
**Model 1**					
Total effect	0.0126	0.0023	0.008	0.017	
Mediation effect of extroversion	0.0038	0.0024	0.0015	0.0065	30.16%
Mediation effect of neuroticism	0	0.0004	−0.0007	0.0009	0
Direct effect	0.0088		0.0043	0.0135	69.84%
**Model 2**					
Total effect	0.0060	0.0015	0.003	0.009	
Mediation effect of neuroticism	0.0037	0.0015	0.0008	0.0067	61.67%
Direct effect	0.0023	0.0021	−0.0018	0.0063	38.33%

## Discussion

To our knowledge, our present work represents the first study to reveal a relationship between Chinese traditional personality traits and Western Eysenck’s personality traits in terms of both theoretical and neurobiological contexts. These results suggest that five-pattern personality traits and Eysenck personality traits may be compatible with one another and that these traits overlapped at the neuronal level to some extent. In TCM, the concept of Yin and Yang—which focused on keeping a balance between the light and dark—explains both the formation of individuals and their nature in terms of Yin and Yang (Yang and Xue, 2006). In general, Yang is a notion linked to openness, extraversion, and enthusiasm. In contrast, Yin often associated with introversion, reticence, and sensitivity. Compared with that of EPQ scores, our result showed that TaiYang and ShaoYang scores were significantly positively correlated with extraversion, which was an expected result. This finding illustrates the interpretation of universality for extroversion, independent of Chinese five-factor personality traits or Western Eysenck’s personality traits. However, a correlation between TaiYang and neuroticism was not found in our study, which seems to contradict the experience that people who score high on TaiYang have more neuroticism and tend to be more impulsive. This result may imply that the emotional impulsivity exhibited by typically TaiYang people does not correspond to the neural stability/instability framework described by Eysenck’s personality theory. TaiYin and ShaoYin scores were significantly negatively correlated with extraversion, and this result was following our expectations. Besides, PingHe scores were significantly associated with all three of the EPQ sub-scales scores. Overall, our results verify previous findings regarding the relationship between FPPI and EPQ scores (Ren, 2014).

In the present study, we found, for the first time, that individuals with high TaiYang scores had significantly increased ReHo scores in the right STG, while the ReHo scores of the right STG showed a significant linear relationship with extraversion and neuroticism scores. These findings may imply a common neural correlate for the two investigated types of personality theories. According to prior studies, the STG is involved in auditory and social cognition processing and plays a role in monitoring reappraisal of behavior (Allison et al., 2000; Singer, 2006; Bigler et al., 2007; Pelphrey and Carter, 2008). Furthermore, the STG is involved in emotional processing (Allison et al., 2000). Within this context, research began to be increasingly concerned about the role of STG in emotional expression and experience. Interestingly, the activation of STG seems to be related to externally oriented behavior characteristics such as extroversion, anxiety, agreeableness, conscientiousness. For example, a task-based fMRI study found that STG activation significantly positively correlated with impulsivity, and a structural study also found that the volume and surface area of the STG significantly positively correlated with agreeableness and anxiety (De Bellis et al., 2002; Leland et al., 2006; Tolkunov et al., 2010; Li et al., 2017). From the TCM perspective, TaiYang represents the extreme form of Yang and is described as one who is outgoing, impulsive, sociable, adventurous, and irritable (Yang and Xue, 2006). The characteristics of the activities for STG that marked by externally-oriented seem to affect the typical patterns of behavior with Taiyang traits. Otherwise, previous fMRI studies on personality have implicated a positive correlation between extroversion, with STG activity during the presentation of emotional stimuli (Mobbs et al., 2005; Tolkunov et al., 2010). This result supports our view that TaiYang and extraversion may overlap not only in theory but also in terms of spatially localized neural activity to a certain degree, and present mediation-analysis results provide further evidence for this association. It is noteworthy, however, that TaiYang did not show any significant correlation to neuroticism scales, and that the linear regression model showed linearity between STG activation and neuroticism. However, our present finding regarding neuroticism seems to contradict previous fMRI studies. There have been reports that task-based fMRI activation in the STG is associated with lower neuroticism scores (Kumari et al., 2007; Coen et al., 2011). Our mediation analysis results also showed no mediating effect of neuroticism between TaiYang traits and brain function. This result is consistent with our scoring results may indicate that there is little correlation between TaiYang and neuroticism, and indicate that TaiYang and extroversion, from different cultural-background personality traits, may be partially engaged in similar mechanisms in the STG.

For TaiYin, we found for the first time that individuals with high TaiYin scores had significant correlations with the ReHo of the mPFC, while the ReHo of this brain area showed a significant linear relationship with neuroticism scores. In recent years, the mPFC has been an active area of focus in the field of social cognition, such as in the theory of mind, emotional recognition, social reasoning, decision making, moral judgment, and self-cognition (Bechara et al., 1996; Adolphs, 1999; Greene et al., 2001; Williams et al., 2005). The activity of mPFC has associated with self-referential processing. The stronger self-referential processing observed in response to negative emotional faces in high neurotic participants (Cremers et al., 2010). Social cognition includes the cognition of the social subject himself/herself (i.e., self-cognition). Neuropsychological and brain-damage research has shown that the development of the PFC is associated with self-awareness and that damage to the mPFC is more likely to lead to a decline in self-cognition (Adolphs, 1999; Anderson et al., 1999; Bar-On et al., 2003). Zhang et al. (2006) used an fMRI block design to explore how individuals make semantic judgments. The results showed that the activation of the mPFC was related to self-cognition. Besides, the results of Johnson et al. (2002) also showed that activation of the mPFC was associated with self-reflection. Further research by Johnson et al. (2006) found that the mPFC was more activated when thinking about self-aspiration and self-obligations. These findings suggest that activation of the mPFC exhibits a self-motivated function. In the present study, the ReHo of the right mPFC was correlated to both TaiYin and neuroticism. Previous studies have shown that the mPFC plays an important role in the neurophysiological basis of narcissistic traits (Moriguchi et al., 2006; Kwan et al., 2007) and meditation experience as primary nodes of the default network (Brewer et al., 2011). TaiYin is the extreme form of Yin and is described as one who is sentimental, narcissistic, jealous, worried, and self-absorbed, according to the theory of five-pattern personalities (Yang and Xue, 2006). People who score high on TaiYin prefer to focus on their emotional experience compared with that of others; therefore, they exhibit a higher degree of introversion. A strong negative correlation between TaiYin and extraversion scores in our study also supports this point of view. Additionally, TaiYin is also related to neuroticism in the field of Chinese personality research. A study based on Chinese college students found that TaiYin was significantly positively correlated with neuroticism (Ren, 2014). Similar results have been found in other studies, suggesting that TaiYin may contain two dimensions comprised of neuroticism and introversion. Furthermore, in our present study, we expectedly found that there was a significant indirect effect of neuroticism within TaiYin and ReHo of the right mPFC. This finding suggests that neuroticism may be a mediator between TaiYin traits and ReHo of the right mPFC. Hence, the relationship between TaiYin traits and neuroticism requires further investigation.

In the present study, we did not find any significant relationships with regional brain function concerning ShaoYang, ShaoYin, and PingHe traits. ShaoYang is a balanced state with more Yang and less Yin. In contrast, ShaoYin is a balanced state with more Yin and less Yang. We think that because of this equilibrium, ShaoYang and ShaoYin did not show a strong extroversion/introversion tendency of any signs of instability. PingHe is a specific conception in TCM that denotes that both Yin and Yang are always in a state of dynamic balance. PingHe represents a model of moderate introversion/extroversion, stability/instability, and flexibility/rigidity. Interestingly, we found that PingHe scores were significantly correlated with the three dimensions of EPQ, especially in terms of a negative correlation with neuroticism and psychoticism scores. This finding indicates that PingHe traits may comprise more of a comprehensive personality. Taken together, we hold the opinion that the theories of Chinese five-pattern personality traits and Eysenck’s personality traits are different in some ways, but they are closely related in terms of describing individual behavioral characteristics. In our present study, only TaiYang and TaiYin were found to exhibit similarities across Chinese five-pattern personality traits and Eysenck’s personality traits in terms of regional brain function. Interestingly, the results of our correlation analysis between these two different cultural personality traits showed that TaiYang and TaiYin might correspond to mixed Eysenck’s traits. Besides, our finding related to extroversion partially mediating the relationship between the right ReHo of STG and TaiYang traits also indicates that the Chinese five-pattern personality traits may offer a more comprehensive explanation for the individual personalities. However, these results need to be taken with more caution as the complexity of personality traits. The simple correlation between two different personality traits and regional brain function offers a useful following study direction—but not enough to clarify a causal interpretation.

Apart from the relatively small sample size, there were some other limitations of our present study. First, our study based on two personality questionnaires, but the quantity and the quality of these samples were not unbiased. The high percentage of females in our study may have influenced our results. One probable cause of the gender division is most of our participants came from nearby universities, mainly liberal arts college where female students are more than the male. Second, we found a significant linear relationship between brain functions and EPQ personality traits; however, the resultant small R-square implied a weak relationship for our regression model. Third, as exploratory research, we only explored the similarity between two theories of personality traits based on different cultural backgrounds at the level of regional brain function. The linear relationship between personality traits and neurological function still can not explain the detailed mechanism working in regional brain function effect on human behavior. Personality is a comprehensive psychological system. Because of its characterization, functional connectivity and network research are the most suitable approaches to study personality in our field. Our present work aims at building a solid foundation for the future of personality research. In further investigations, we will adopt a multimodal neuroimaging method to construct a neural personality model based on specific cultural backgrounds.

In conclusion, we used regression and mediation analysis to explore the relationship between two cultural theories of personality traits from the perspective of theoretical construction and neuroimaging. We found that the ReHo of the right STG, which showed a significant linear relationship to extraversion and neuroticism, was positively correlated with TaiYang. Extroversion also played a mediating effect between TaiYang and ReHo of the right STG. In addition, the ReHo of the right mPFC, which showed a significant linear relationship to neuroticism, was positively correlated with TaiYin. Our findings suggest that Chinese five-pattern personality traits may be associated with Eysenck’s personality traits and that Chinese five-pattern personality traits may have a broader and deeper neurobiological basis compared to that of Eysenck’s personality traits. We conclude that both theories of personality traits may be represented by similar or parallel neural mechanisms within function-specific brain regions. However, this study only is exploring the linear correlation between personality traits and neurological function. The detailed mechanism working in regional brain function effect on two traits from different cultures should be further explored.

## Data Availability Statement

The datasets generated for this study are available on request to the corresponding author.

## Ethics Statement

The studies involving human participants were reviewed and approved by Ethics Committee of the Shanxi Medical University. The patients/participants provided their written informed consent to participate in this study.

## Author Contributions

YX and QY designed and supervised the study. WZ and LS drafted the manuscript. JD, XRL, HW, LZ, and LC carried out the experimental procedures. WZ, JL, and XZL undertook the statistical analyses and reviewed the literature.

## Conflict of Interest

The authors declare that the research was conducted in the absence of any commercial or financial relationships that could be construed as a potential conflict of interest.
